# Mentha-Stabilized Silver Nanoparticles **for High-Performance Colorimetric Detection of Al**(III) in Aqueous Systems

**DOI:** 10.1038/s41598-018-23469-1

**Published:** 2018-03-26

**Authors:** Rekha Sharma, Ankita Dhillon, Dinesh Kumar

**Affiliations:** 1 Department of Chemistry, Banasthali University, Banasthali, Rajasthan 304022 India; 2 School of Chemical Sciences, Central University of Gujarat, Gandhinagar, 382030 India

## Abstract

The present paper reports a facile and selective colorimetric method for the detection of potential environmental and health hazardous metal ions using green synthesized silver nanoparticles (AgNPs). Here the organic functional groups present in the plant extract (*Mentha arvensis*) are used as reductants and stabilizers in the synthesis of AgNPs. They also provide a suitable binding site to the (Al(III)) analyte in the detection mechanism. The leaf extract of *Mentha arvensis* was used to synthesize AgNPs at room-temperature and at 80 °C. The AgNPs synthesized at 80 °C exhibit excellent selective colorimetric detection of Al(III). The as-synthesized AgNPs have been characterized, and the synthesis, stabilization of NPs and detection mechanism has also been illustrated by using UV-vis, XPS, FTIR, TEM, EDX, SEM, AAS, and TGA analytical tools and techniques. The selectivity of detection probe was supported by the reaction between probe and metal ions followed first-order kinetics having the highest value of the regression coefficient (R^2^ = 0.99) for Al(III) and the analysis of thermodynamic parameters. The prepared sensor showed a lower limit of detection (LOD) of 1 nM (S/N = 3.2) in real water samples. The proposed method can be successfully utilized for the detection of Al(III) from both drinking and real water samples at the nanomolar level.

## Introduction

Water contamination due to various metal ions such as Al(III), Hg(II), Cd(II), Pb(II), As(III), Mn(II), Cr(III), and Cr(VI) is a global problem^1,2^. Aluminum is the third most abundant element in the earth’s crust and it has wide applications in the field of aliment additives, pharmaceuticals and production of cookware^3–5^. Overconsumption of aluminum causes damage to the nervous system^6^, idiopathic Parkinson’s disease^7^, memory impairment^8^, Alzheimer’s disease^9^, and dialysis encephalopathy^10^. In plasma, Al(III) is carried by the iron-binding protein and therefore, it can easily penetrate the brain and can even reach the placenta and fetus. The concentration of aluminum in the brain should not cross the 2 mg/g^11^. Much research has been done on the detection and the removal of such toxicants or analytes from drinking water. The Environmental Protection Agency (EPA, USA) and World Health Organization (WHO) recommend a maximum limit of Al(III) contamination as 50 ppm and 7.4 μM in drinking water, respectively^12,13^. Thus, the ability to detect significantly low concentrations of Al(III) is vital for agriculture, industry and human health. The Al(III) has been detected using atomic absorption spectrometry (AAS)^14^, inductively coupled plasma-atomic emission spectrometry (ICP-AES)^15^, inductively coupled plasma-mass spectroscopy (ICP-MS)^16^, graphite furnace- atomic absorption spectrometry (GF-AAS)^17^, and electrochemical methods^18^. Although these methods have high sensitivity, unfortunately, these methods are not always reliable and are limited by their expensive nature, tedious sample treatment methods and production of high background signals^19^. Hence there is an urgent need for a commercially viable, economical, rapid, and environmentally friendly route for the detection of Al(III). The optical, chemical, mechanical and electrochemical properties of noble metal nanoparticles make them an excellent resource for varied applications in diverse fields - anticancer^20^, cosmetics^21^, coating^22^, and biocatalysis^3,4^. Recently, colorimetric assays and nanoprobes such as paper-based analytical device (PAD), quantum-dots-based photoelectrochemical immune assays, magnetic bead-based reverse colorimetric sensors, MnO_2_ nanoflakes with enzyme cascade amplification for colorimetric immunoassay, Fenton reaction-based colorimetric immunoassay for sensitive detection of brevetoxin B have been synthesized for the colorimetric detection of metal ions in aqueous system^23–28^. Aggregation of NPs leads to a visual color change that is important for the detection of analyte without complex instruments^29^. Therefore, aggregation mechanism has been utilized to detect several metal ions by using NPs and nanocomposites by our research group^30–32^. As compared to AuNPs, AgNPs are more suitable for detection applications because of ~100-fold greater molar extinction coefficient of AgNPs, resulting in higher sensitivity in absorption spectroscopy^33^. In this research, we have developed a green detection method for Al(III) with 0.01 ppm lower detection limit by necked eyes^17^. Though several reports have been published on the colorimetric detection of Al(III) ions^32–35^, to date, to the best of our knowledge, no work has been carried out on the detection of Al(III) with 1 nM level using green synthesized AgNPs in an aqueous medium.

In continuation of our research interest in the development of more efficient methods, we have studied the application of AgNPs functionalized with the leaf extract of Mentha (M-AgNPs) for the highly selective and sensitive detection of aqueous Al(III) with 1 nM level. The plant extract of Mentha arvensis contains flavonoids and the phenolic compound as a major component which may be involved in the bioreduction of Ag(I) to Ag(0). The flavonoids have the good reducing capacity, therefore the moment they come in contact with Ag(I) ions, they transfer their *π*-electrons and reduce the Ag(I) to Ag(0). Further, they also help in preventing agglomeration thereby stabilizing the AgNPs^36^. Therefore, the flavonoids play a dual role by rapidly reducing Ag^+^ ions and as a capping agent, which gives stability to AgNPs. This paper introduces a sustainable method which rectifies the low detection limit of the previous work. The present study also aims to detect Al(III) with a visual color change as compared to other environmentally relevant metal ions. The detection of Al(III)ion in aqueous samples has been achieved within one min. Further, the Al(III) ions’ interaction with M-AgNPs has been established by X-ray Photoelectron Spectroscopy (XPS), Fourier Transform Infrared Spectroscopy (FTIR), Zeta potential and Energy Dispersive X-ray (EDS) spectroscopy studies.

## Results and Discussion

### Synthesis mechanism of M-AgNPs

**Figure 1:**
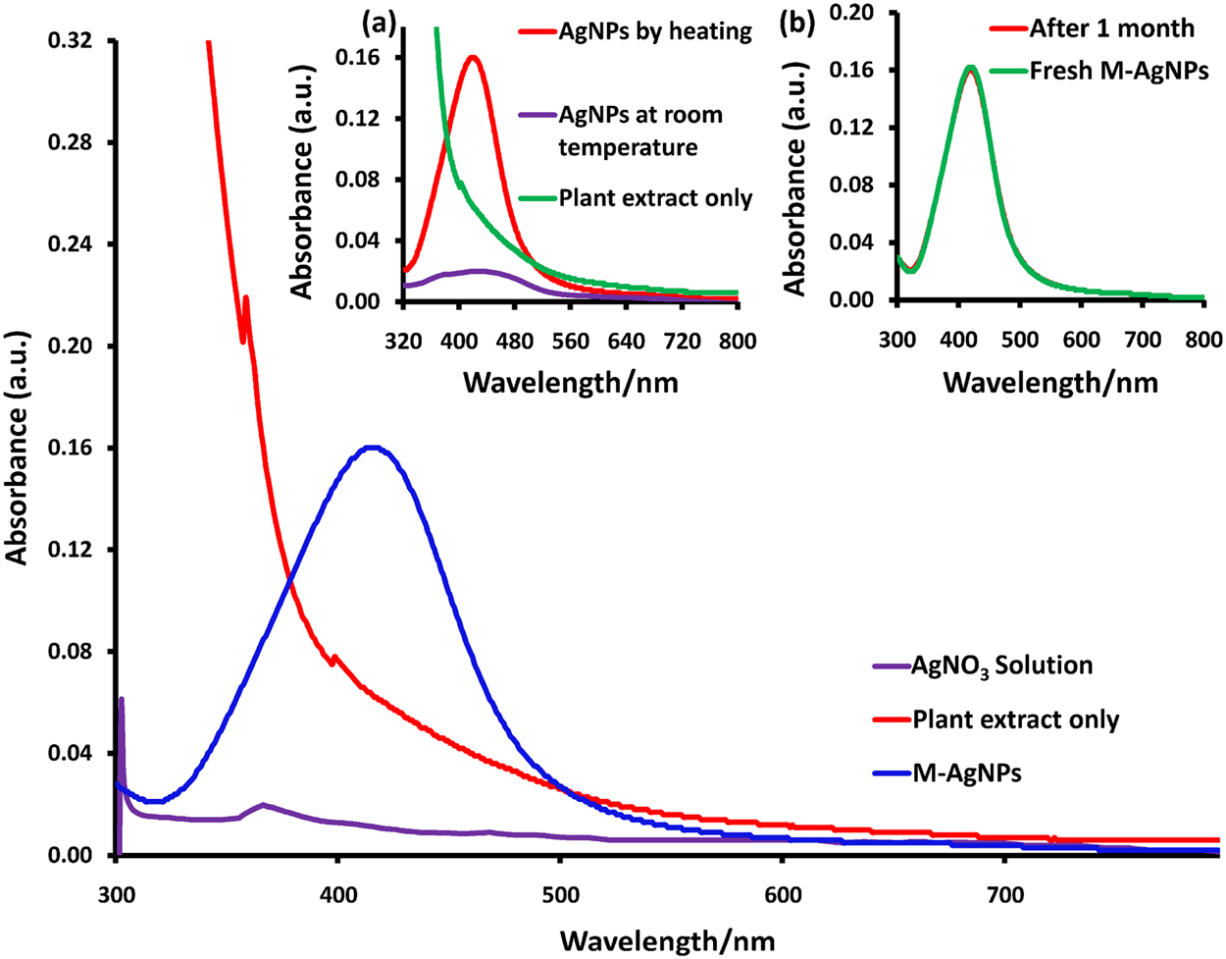
UV-vis absorption spectra of the AgNO_3_ solution, MLE, and M-AgNPs, inset shows (**a**) M-AgNPs prepared by the heating method and at room-temperature, (**b**) stability of M-AgNPs after 1 month.

**Figure 2:**
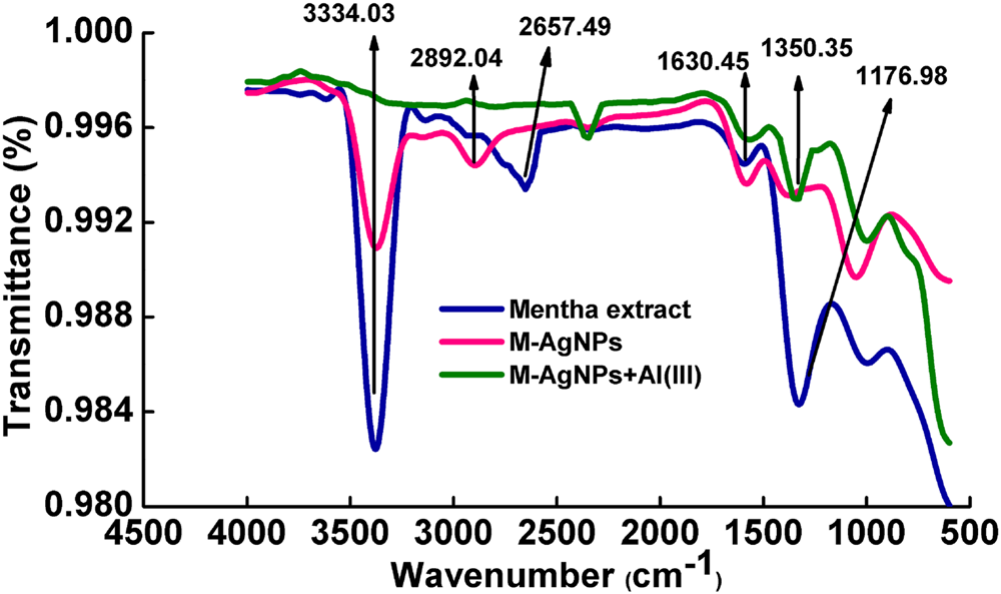
FTIR spectra of MLE, M-AgNPs, and M-AgNPs with Al(III).

A characteristic Surface Plasmon Resonance (SPR) peak appears at 419 nm, corresponding to the dark yellow solution of M-AgNPs (Fig. 1a) that indicates the formation of M-AgNPs. Many small bands in the IR spectrum of Mentha are the common feature of small molecules. The stretching vibrations due to the aromatic ring of Mentha were obtained at 1630.45 cm^−1^; a single absorbance peak located at 1176.98 cm^−1^ is assigned to C-O polyols; a peak around 2657.49 cm^−1^ corresponds to carbonyl (C=O) group while broadband at 3334.03 cm^−1^ corresponds to the O-H group. After the interaction of AgNO_3_ solution with Mentha extract, redshifts occurred at 2892.04 cm^−1^ confirming the formation of M-AgNPs. The absence of these bands in the IR spectrum of M-AgNPs confirms the involvement of carbonyl groups in the reduction step. Additionally, the appearance of new bands in the IR spectrum of M-AgNPs in the region of 1350.35 cm^−1^ corresponds to C-O-H deformation and 2322.40 cm^−1^ confirm the formation of M-AgNPs. The absence of O-H group in the FTIR of M-AgNPs with Al(III) confirms the binding of Al(III) with M-AgNPs (Fig. 2)^37^.

### Stability of M-AgNPs

The stability of the detection system over a range of conditions is of immense significance in colorimetry. The stability of M-AgNPs was estimated by UV-vis spectroscopy in terms of pH, the volume of MLE, temperature and time. Further, the zeta potential was measured to assess the stability and solubility of AgNPs in aqueous solution. The negative zeta potential value of −41.8 mV at pH 10.5 indicates excellent stability without any sign of coagulation or precipitation for months.

### Effect of pH

**Figure 3:**
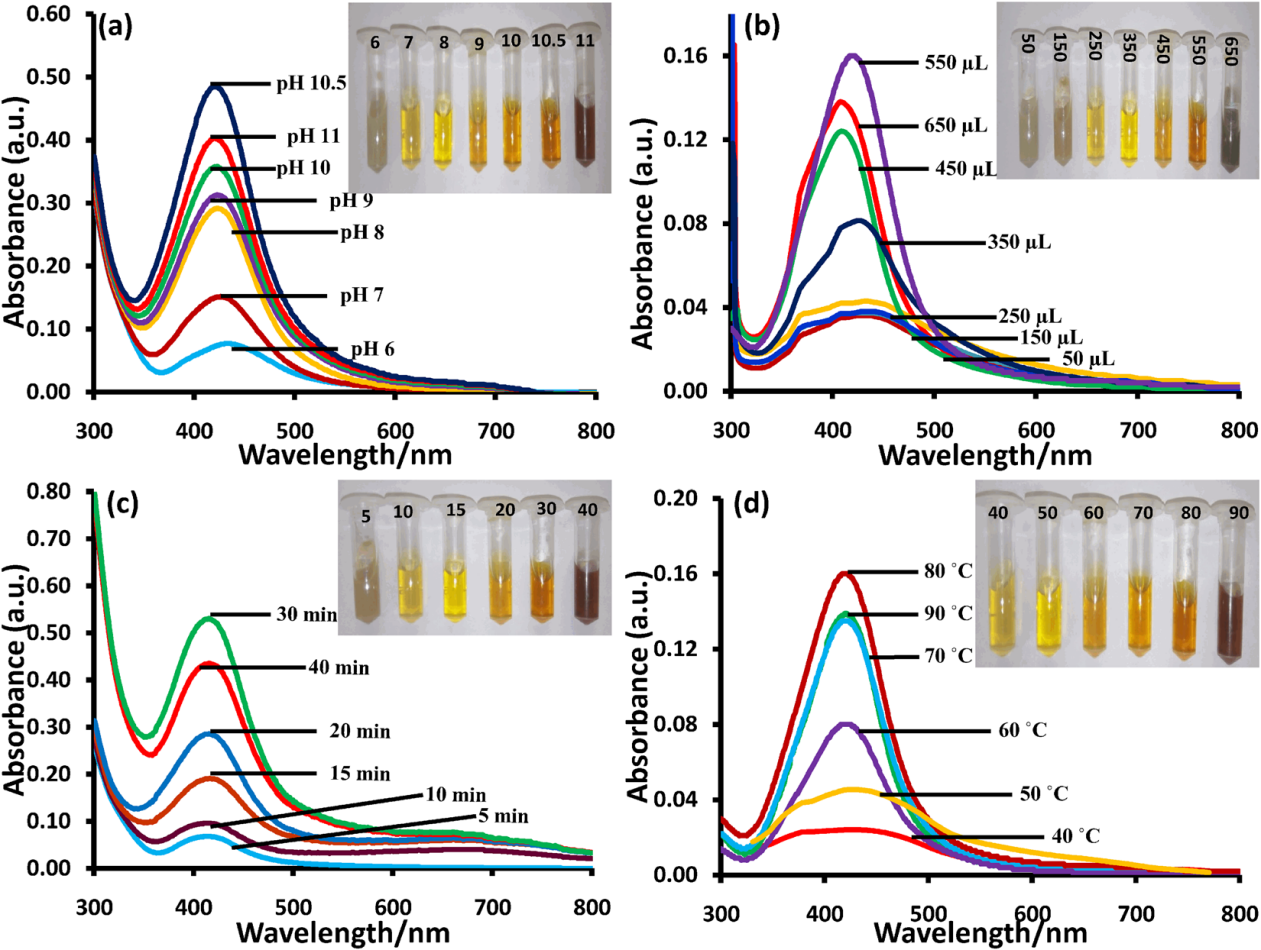
UV-vis spectra of the M-AgNPs prepared at different (**a**) solution pHs, the inset shows colorimetric change at pH, (**b**) MLE volumes, the inset shows the photographs of the corresponding solutions, (**c**) time intervals, and (**d**) temperatures, the inset shows the photographs of corresponding solutions.

The stability of nanoparticles in a dynamic medium depends on various factors such as size and shape of NPs, the charge on NPs, the dielectric constant of the medium, pH etc. Hence, herein the stability of NPs was studied at a wide range of pH 6–11 (Fig. 3a). Inset of Fig. 3a shows variations in color of M-AgNPs solution at different pH values (6–11). Subsequently, a dark brown color was observed due to the aggregation of M-AgNPs upon increasing the pH from 10.5 to 11. A continuous increase in the intensity and sharpness of the SPR band with a blue shift from 425 to 419 nm was found at pH range 6 to 10.5. These results confirm the highest stability of M-AgNPs at pH 10.5. The intensity and sharpness of peak decreased upon increasing the pH from 10.5 to 11. Therefore, further experimentation was done at absolute pH 10.5.

### Effect of extract volume

The stability of M-AgNPs was also studied as a function of the volume of MLE. For this, varying volumes of MLE (50–650 μL) were added to different AgNO_3_ solutions for the reduction of the AgNO_3_ solution. The visual color change in AgNO_3_ solution from gray to yellow was observed upon varying the volume at 550 μL (inset of Fig. 3b). To confirm these observations, SPR spectra were taken, and the intensity of the peak was increased by increasing the volume of MLE from 50 to 550 μL. Upon changing the volume from 550 to 650 μL, an instant color change from yellow to dark gray was observed with decrease in intensity and increased peak broadening. The full width at half maxima (fwhm) of the corresponding peaks determines the dispersity of nanoparticles, where a large fwhm was attributed to peak broadening due to increased polydispersity of nanoparticles. As the size of particles increased, fwhm values increased from 96 to 104 nm (Fig. S1a and b).

Therefore, 550 μL of MLE was chosen as the optimal volume to stabilize M-AgNPs.

### Effect of reaction time

The reaction time was increased from 5 to 40 min. During this time variation, the colorless solution of AgNO_3_ started to darken (Fig. 3c inset). Figure 3c shows that no significant optical absorption occurred in the range of 400–500 nm, only a shoulder was observed at around 430 nm after 5 min. On further increasing the reaction time, the shoulder started converting into a peak at 419 nm, which became sharpest at 30 min. On further increasing the reaction time from 30 to 40 min, the peak intensity decreased with peak broadening, and the color of the solution changed from yellow to dark brown (Fig. 3c inset). As the size of particles increased, fwhm values increased from 98 to 119 nm (Fig. S2a and b). Therefore, 30 min reaction time was chosen as the optimal time to stabilize M-AgNPs.

### Effect of temperature

The effect of temperature on the stability of M-AgNPs was studied by varying the synthesis temperature from 40 to 90 °C. At 40 °C, SPR spectra do not show a peak in the region of 400–500 nm (Fig. 3d). The intensity of a featured peak was increasing with the temperature, which eventually reached a maximum at 80 °C. The intensity of the peak was decreased afterward, hence, 80 °C was set as an optimum temperature. Consequent color variation in M-AgNPs solution was observed (Fig. 3d inset).

### Characterization of M-AgNPs

**Figure 4:**
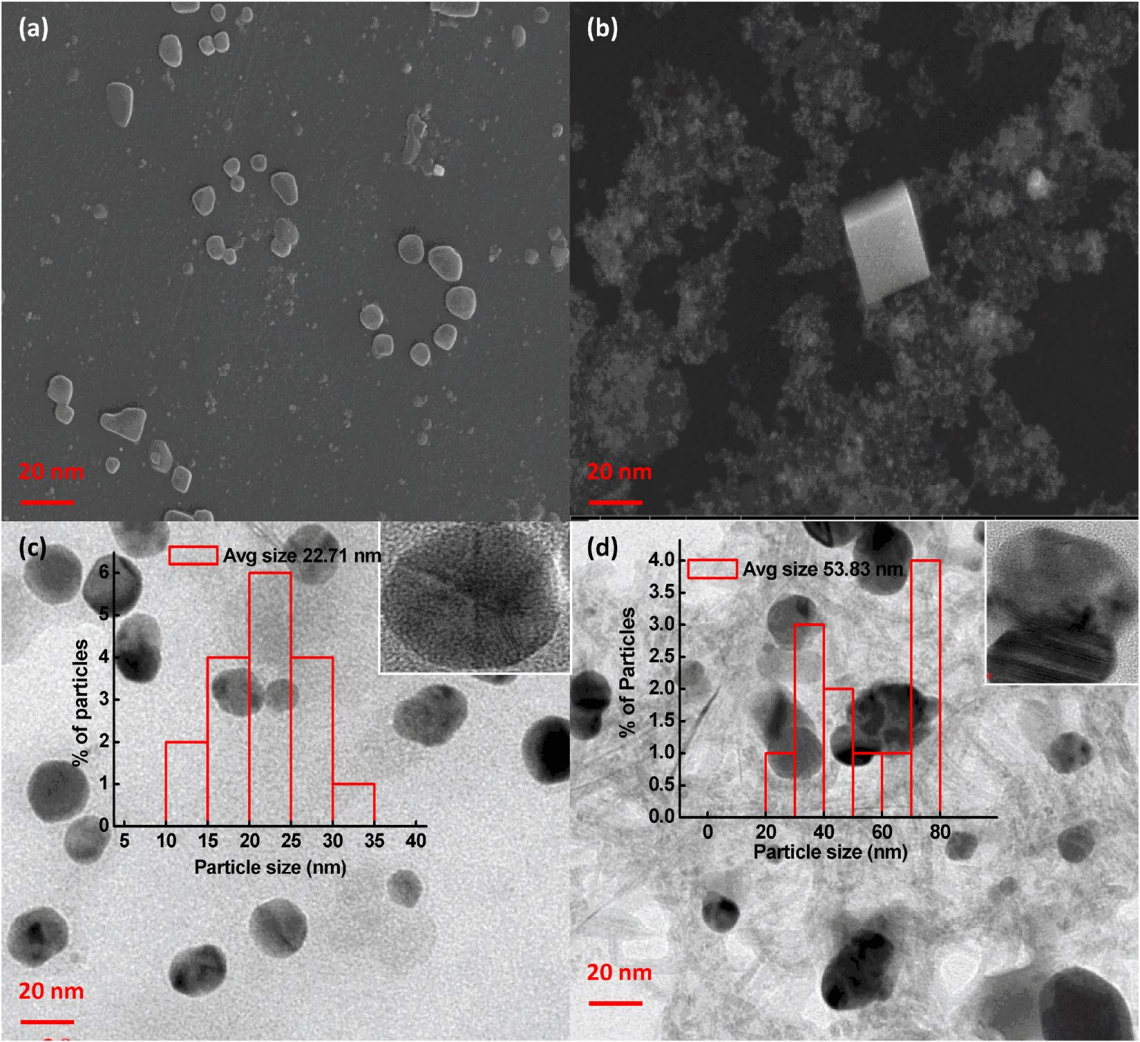
(**a**) SEM image of M-AgNPs before and (**b**) after interaction with Al(III), (**c**) TEM image of M-AgNPs before and (**d**) after interaction with Al(III).

**Figure 5:**
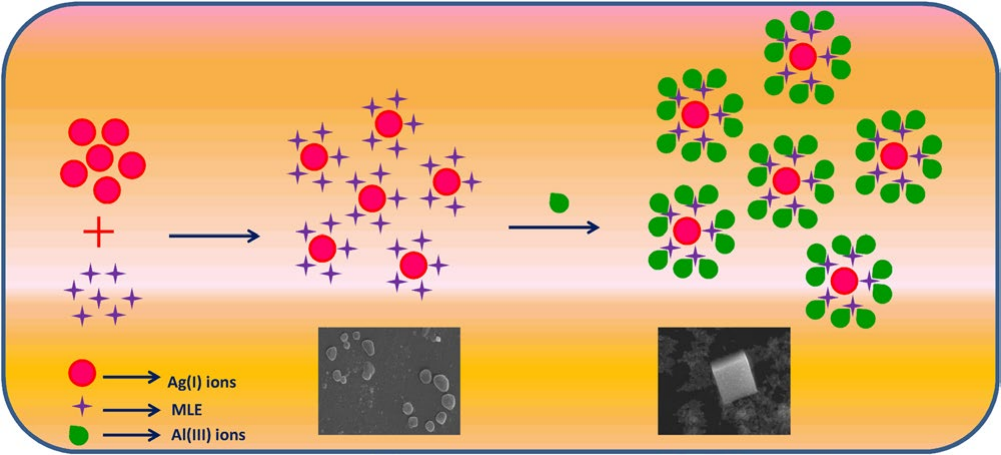
Schematic representation of synthesis and Al(III) induced aggregation of M-AgNPs.

SPR spectra of MLE and AgNO_3_ showed no characteristic peak in the region of 400–500 nm (Fig. 1). The appearance of a new SPR band at 419 nm at pH 10.5 confirms the reduction of Ag(I) ions into Ag(0) and formation of M-AgNPs by MLE. The surface morphology and size of M-AgNPs were studied by SEM and TEM images at pH 10.5 (Fig. 4). The average particle size was 22.71 nm at pH 10.5. The size was increased (53.83 nm) after interaction with Al(III) due to agglomeration caused by these metal ions. These results are well agreed with the results of SPR and zeta sizer studies (Fig. 1 and S3). TEM and SEM images show well dispersed oval shaped particles. The increased charge accumulation on the M-AgNPs surface with pH is ascribed to the electrostatic stability of particles^38^. A mechanistic pathway of the synthesis of M-AgNPs is presented here based on the present study (Fig. 5). Flavonoids and menthol are the major constituents of MLE.

### Thermogravimetric analysis (TGA), Energy dispersive X**-**ray spectroscopy (EDS) and Selected area electron diffraction (SAED) studies

**Figure 6:**
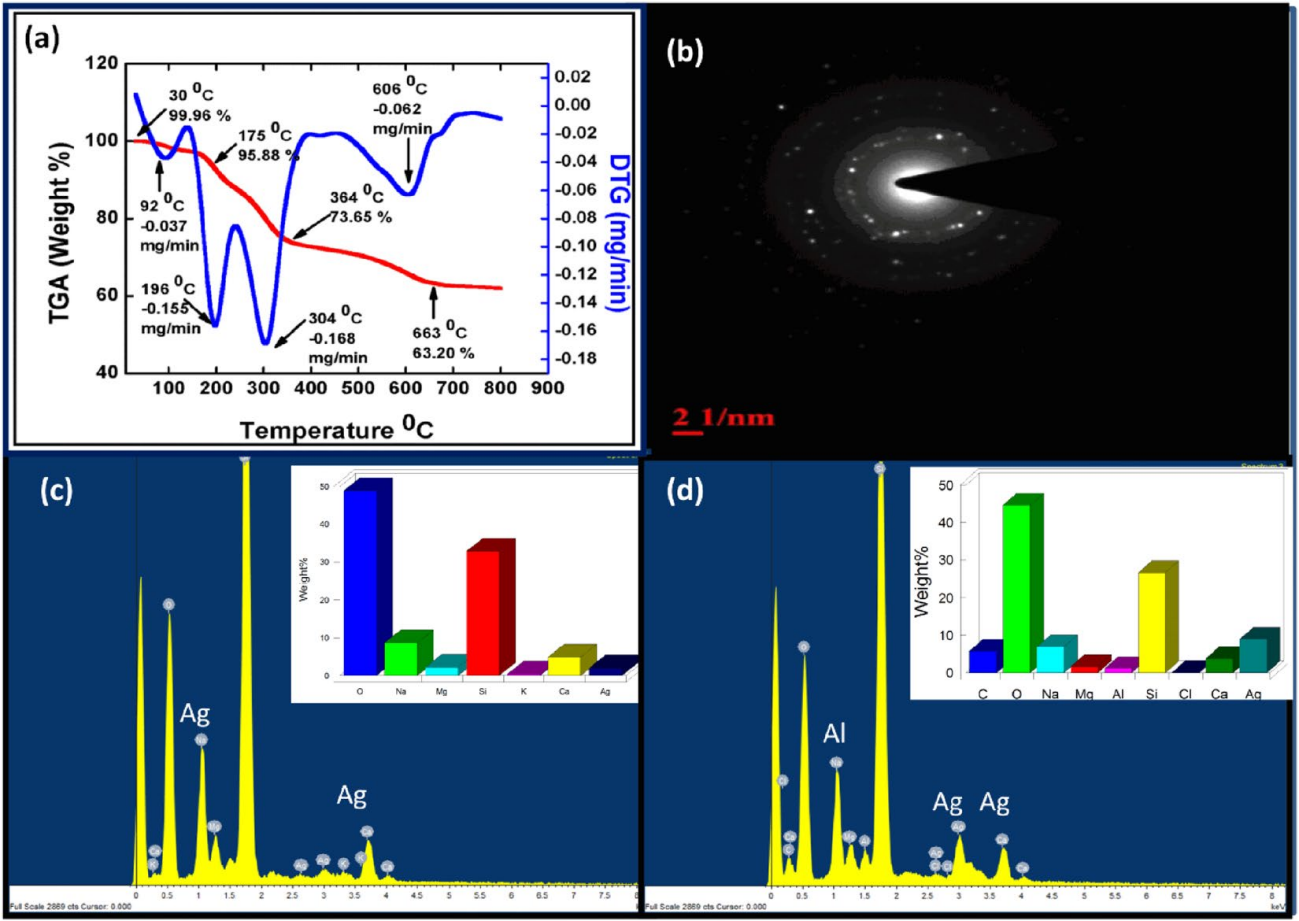
(**a**) TGA, (**b**) SAED, (**c**) EDX analysis of M-AgNPs before and (**d**) after interaction with Al(III).

The thermal studies of the M-AgNPs were done using TGA analysis (100 to 800 °C). The thermogram indicates that the initial weight loss in the range of 100–200 °C, which may be due to loss of water molecules present in the nanoparticles. Further weight loss occurred within 100–750 °C. The total weight loss of 37.20% may be because of desorption of the surface-active components present in the MLE^39^. These results demonstrate the vital role of the Mentha biomolecules in the nucleation, growth, and stabilization of AgNPs (Fig. 6a). Thermal stability of M-AgNPs is directly dependent on the decomposition temperature of its various functional groups. The TGA and DTG curves of M-AgNPs are shown in Fig. 6a. The TGA curve shows three main zones, in which the first zone, from 30 (99.96%) to 175 °C (95.88%) represents the loss in weight of M-AgNPs due to moisture and light volatile compounds. The second zone is from 175 to 364 °C (73.65%) in which weight loss of M-AgNPs increases due to the evolution of CO_2_ and CO at a high rate. The third zone is from 364 to 663 °C (63.20%) where weight loss slightly declined and maintained near to constant values. The obtained total weight loss in TGA curve is 37.20%. The maximum degradation rates are found to −0.037, −0.155, −0.168, and −0.062 mg min^−1^ at 92 °C, 196 °C, 304 °C, and 606 °C, respectively for M-AgNPs in DTG curve.

A SAED pattern of M-AgNPs was recorded by directing the electron beam perpendicular to one of the individual particles. The characteristic bright circular fringes can be indexed to (111), (200), (220), and (311) of the pure face centered cubic (FCC) lattice structure (Fig. 6b). The elemental composition of M-AgNPs was studied by means of EDS analysis. The presence of Ag peak confirms the formation of AgNPs (Fig. 6c). In Fig. 6d, the presence of Al peak confirms the interaction of Al(III) metal ion with M-AgNPs.

### Selectivity of the detection probe

The ligands having oxygen and nitrogen moieties have high metal ions affinity^40^. Therefore, we chose MLE to synthesize M-AgNPs. All the selectivity experiments were conducted in triplicate.

**Figure 7:**
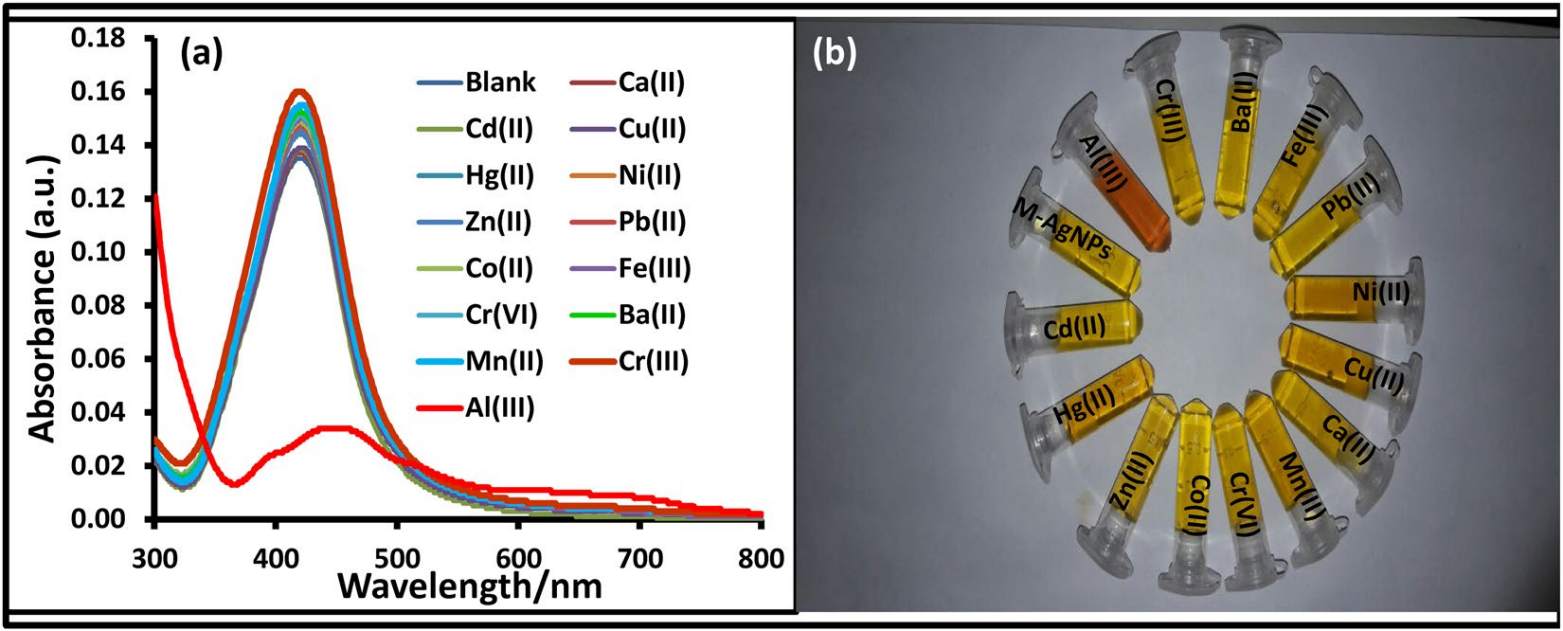
UV-vis spectra of the M-AgNPs upon addition of different metal ions, (**b**) visual color change of the M-AgNPs with various metal ions.

SPR spectra showed a new peak at 462 nm upon addition of Al(III), while no noteworthy change in SPR spectra was observed in the presence of other metal ions (Fig. 7a). Although M-AgNPs were stable at room-temperature, their aggregation occurred in the presence of Al(III) ions while no or negligible interference was noticed in the presence of other metal ions. Further, the selectivity of M-AgNPs towards Al(III) was done by plotting the absorption intensity ratio (A_462_/_419_) of M-AgNPs against metal ions concentration (Fig. S4). The value resulting from Al(III) interaction was the highest thus establishing the characteristic interaction of Al(III) with M-AgNPs. The selectivity of the detection probe was further checked on various concentrations (10–100 nM) of different metal ions which also showed the same results (Fig. S5a). These outcomes due to the interaction between Al(III) and M-AgNPs were further confirmed by a change in color of the solution. Upon addition of Al(III) ions, the dark yellow solution of M-AgNPs changed to reddish-brown due to interaction with Al(III) ions. On the other hand, the color of M-AgNPs remained unaltered with other metal ions (Fig. 7b). It is evident from Fig. S5b that there were significant optical changes for Al(III) ions, irrespective of the presence of other cations. The results demonstrate that the interaction of M-AgNPs with Al(III) ions are unaffected by the presence of other metal ions.

Further, the SEM images of M-AgNPs in the presence and absence of Al(III) ions show the interaction of Al(III) with M-AgNPs altered the shape from oval to cubic (Fig. 4a and b). This morphological transition signifies ionic interactions with Al(III)^41^.

### Sensitivity of the detection probe

**Figure 8:**
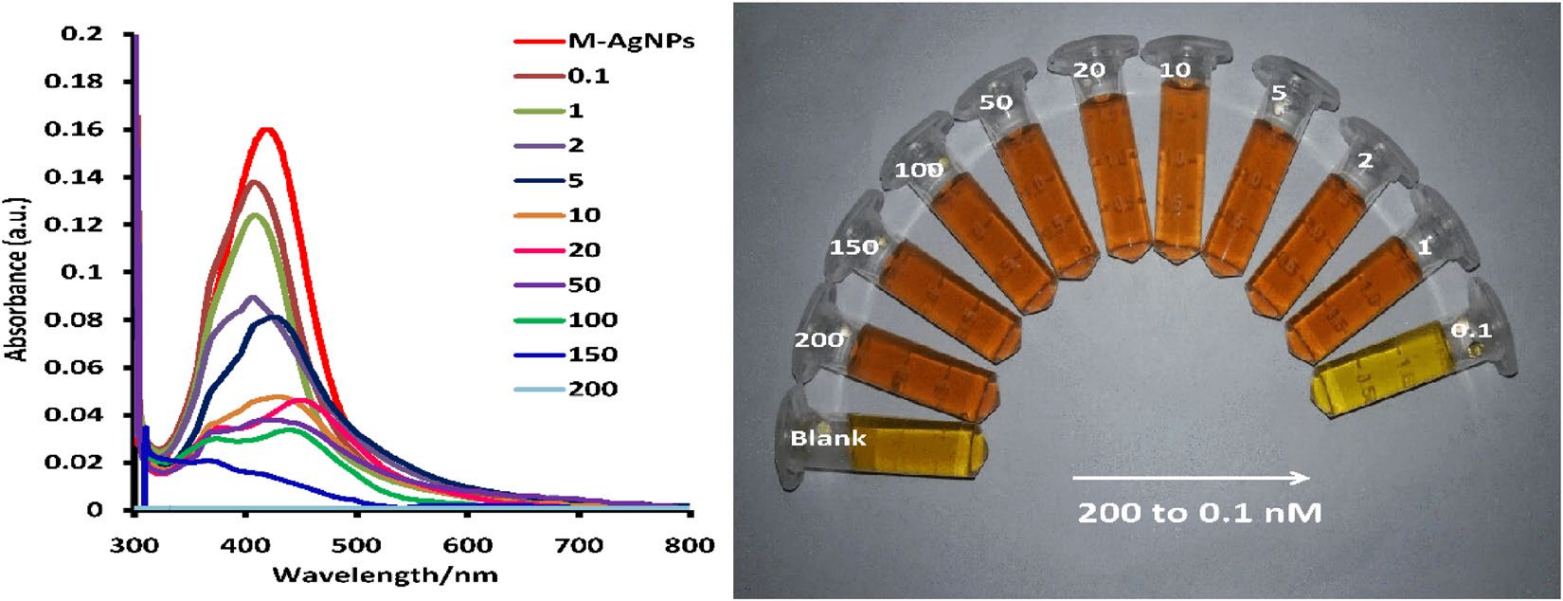
(**a**) UV-vis spectra of the M-AgNPs upon addition of different Al(III) ion concentrations, and (**b**) visual color change of the M-AgNPs with various Al(III) ion concentrations.

Sensitivity studies were conducted by interacting different concentrations of Al(III) with M-AgNPs, and the alterations in the peak intensity, width, and position were studied by UV-vis spectroscopy. Solutions of different concentrations were prepared to take SPR spectra. The SPR peak intensity at 419 nm reduces with Al(III) ion concentration (Fig. 8a). The color of the solution having Al(III) ion concentration of 0.1 nM served as control. The yellow solution of M-AgNPs changed to reddish-brown with increasing concentration of Al(III) ion (Fig. 8b). This visual color alteration in M-AgNPs solution could be due to functional group interaction present on the surface of NPs with Al(III) by metal-ligand interaction. This metal-ligand interaction with Al(III) ions might be responsible for the aggregation of M-AgNPs. The dose-response of the assay was further studied by plotting A_462/419_ values against different Al(III) concentrations (Fig. S6). The plot of Al(III) concentrations ranging from 0.1 to 200 nM presents a linear correlation with linear regression coefficients (R^2^) value of 0.991 (Fig. S6).

The sensitivity of the developed detection probe is 1 nM (S/N = 3.2) for the naked eye, thereby making it an efficient colorimetric sensor for Al(III) in aqueous systems at the nanomolar level. Additionally, no alteration in the characteristic peak of nanoparticles in UV-vis spectra beyond 0.1 nM of Al(III) was observed, which confirmed the above outcomes^42^.

The HRTEM images of M-AgNPs in the presence and absence of Al(III) ions show the monodispersible nature of M-AgNPs which aggregate on the addition of Al(III) (Fig. 4c and d).

MLE is composed of flavonoids and phenolic compounds, such as menthol, menthone, pulegone etc^43^. These phenolic hydroxyl constituents have high affinity towards Al(III) ions^44^. The smaller size and higher effective nuclear charge of Al(III) ion provide a good binding site as compared to other metal ions. Al(III) is a hard acid and it tends to coordinate preferably with a hard base such as N and O atom^45^. Consequently, it can easily coordinate with the negatively charged oxygen of polyphenols. This type of interaction results in a reduction in the zeta potential of M-AgNPs from −41.8 to 2.0 mV (Fig. S7), which shows strong binding of Al(III) with M-AgNPs. This type of interaction of M-AgNPs with Al(III) results in almost complete neutralization of the nanoparticles’ surface and this results in reduced interparticle distance to induce aggregation of M-AgNPs.

**Figure 9:**
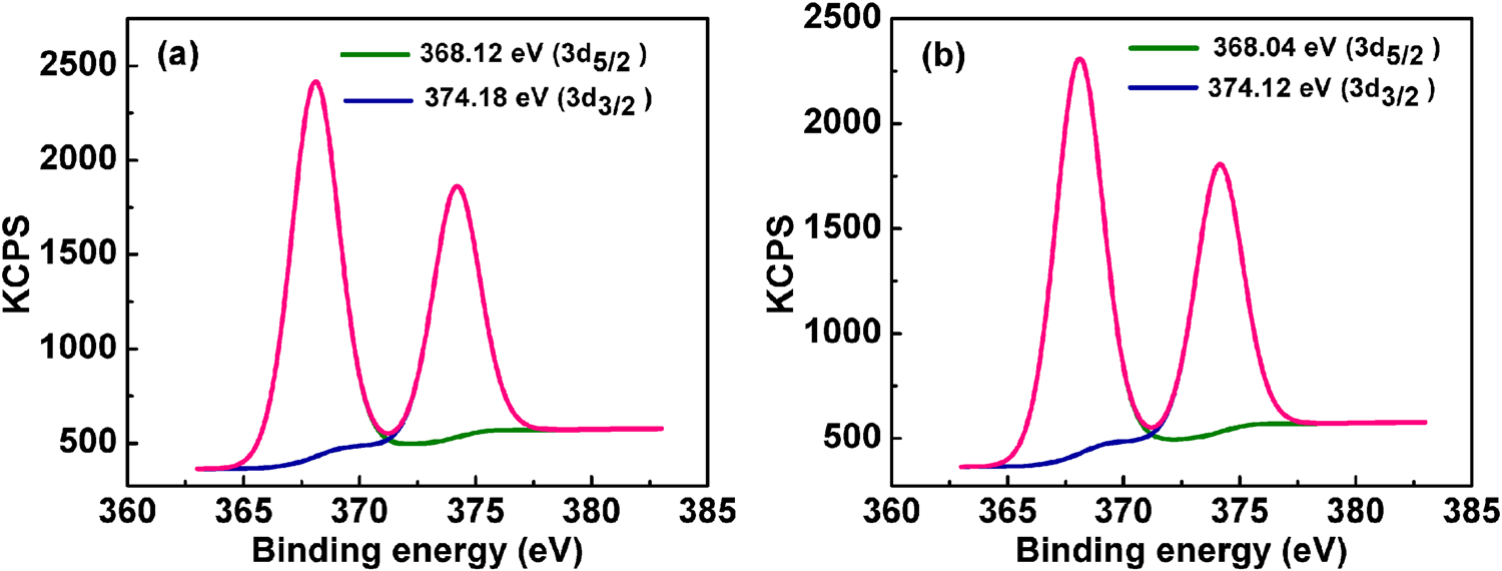
XPS spectra of the green synthesized silver nanoparticles of (**a**) Ag 3d before, and (**b**) Ag 3d after addition of Al(III) ions.

**Figure 10:**
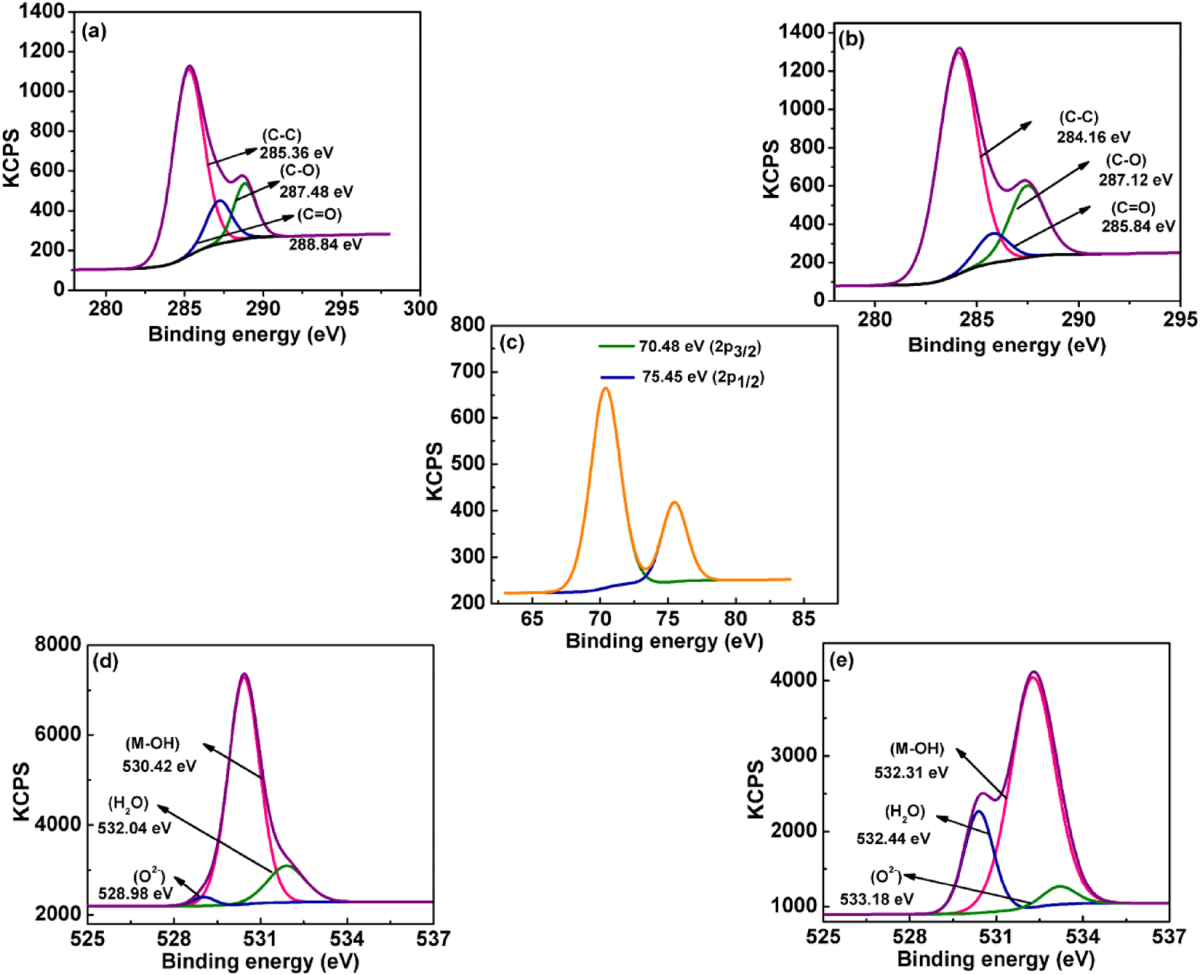
XPS spectra of (**a**) C 1 s before; (**b**) C 1 s after addition of Al(III); (**c**) Al 2p XPS spectra; and (**d**) O 1 s before; (**e**) O 1 s after addition of Al(III).

XPS analysis was used to investigate the binding of Al(III) ions with M-AgNPs (Fig. 9 and 10) to confirm high sensitivity and selectivity. Before the addition of Al(III) ions, the signals at binding energies of 368.12 eV and 374.18 eV were corresponding to the 3d_5/2_ and 3d_3/2_ orbits of Ag(0) (metallic silver) (Fig. 9a)^46,47^. However, after the addition of Al(III), the Ag 3d peaks appeared at the binding energies of 368.04 eV for 3d_5/2_ and 374.12 eV for 3d_3/2_, small binding energy shifts, and slight peaks broaden with respect to metal peaks, indicating the formation of silver oxide (Ag_2_O) after interacting the nanoparticles with Al(III) ions (Fig. 9b). Strong peaks located at about 70.48 eV and 75.45 eV in Al 2p spectrum corresponded to Al(OH)_3_ and Al metal ion, respectively. These peaks confirm the binding of aluminum on the surface of M-AgNPs (Fig. 10c)^48^. The O 1 s spectrum was recorded for Mentha-capped AgNPs and the Mentha-capped AgNPs in the presence of Al(III) ions. The peak at 530.42 eV was assigned to oxygen in the hydroxyl groups of Mentha, the peak at 528.98 eV was assigned to O^2−^, and the peak at 532.04 eV was assigned to H_2_O (Fig. 10d)^49^. After addition of Al(III) ions to the Mentha-capped AgNPs, O 1 s peaks shifted towards higher binding energy which is 532.31 eV, 533.18 eV and 532.44 eV, respectively (Fig. 10e)^50^. The C 1 s spectrum can be deconstructed into three components at about 285.36, 287.48, and 288.84 eV. The first component at a binding energy of 285.36 eV is attributed to the nonoxygenated ring carbon (C-C), and the other two components are attributed to the carbon in C-O (287.48 eV), and the carbonyl carbon (C = O, 288.84 eV), respectively (Fig. 10a). These binding energies are decreased after the addition of Al(III) to 284.16 eV, 287.12 eV, and 285.84 eV, respectively (Fig. 10b)^51^. The change in the binding energy was attributed to the formation of Al–O bonds between M-AgNPs and Al(III) ions. The result provides a strong evidence that the binding of Al(III) ions to the silver nanoparticles is caused by chelation of Al(III) ions on the hydroxyl groups of M-AgNPs. UV-vis spectral changes, HRTEM images, FTIR spectra, XPS, and zeta potential studies confirm the possible detection mechanism of Al(III) ions as shown in Fig. 5.

### Detection of Al(III) in tap water

**Figure 11:**
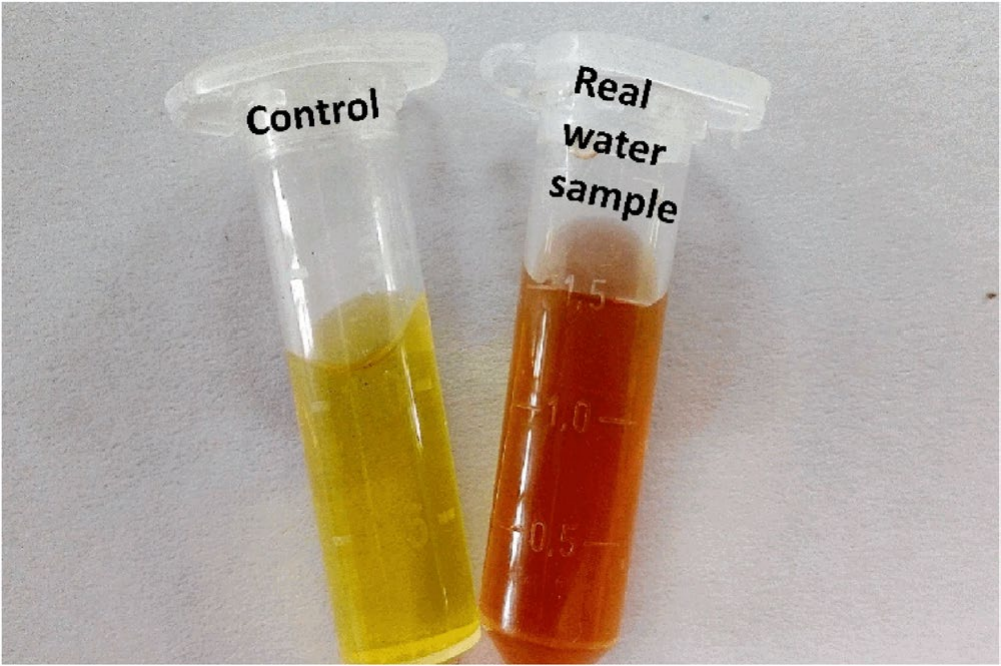
M-AgNPs colorimetric response as the function of Al(III) ion concentration in tap water samples.

To check the field applicability of our detection method, the developed probe was tested on a tap water sample collected from our lab. Since the contamination by Al(III) in the tap water sample was lower than the limit of detection (LOD) of the developed sensor, the tap water sample was further contaminated with standard solutions of Al(III) ion (Fig. 11). The identical response of the developed sensor towards both Milli-Q water and tap water implies the easy detection of Al(III) in tap water lacking interfering contaminants. Therefore, this confirms the utility of the developed nanosensor for the detection of Al(III) ions in real water samples. The sensitivity of this colorimetric sensor was compared to reported methods using nanoparticles as sensors^34,52–55^. The limit of detection of our proposed method is lower or comparable to the other reported methods for Al(III) ion (Table 1). The lower detection limit at nM level, short response time (within 1 min), working at physiological pH (pH 7.4), and applied to the real water samples makes the present method more advantageous over other reported methods^29^.

**Table 1:** Comparative study of various reported sensors with present work in terms of reducing and stabilizing agent, pH, and limit of detection.

Method	Detection probe	Reducing agent	Stabilizing agent	Sample matrix	LOD (nM)	pH	Ref.
Colorimetric	AuNPs	Citrate	MMT*	Water and urine samples	14.29	8.0	^42^
Colorimetric	AuNPs	Citrate	Citrate	water	26	2.9	^43^
Colorimetric	AuNPs	NaBH_4_	Ionic liquid	Vermicelli	26	—	^44^
Fluorescent	Chalcone based organic nanoparticles	—	—	Lake and tap water	29	7.0	^45^
Colorimetric and fluorescent	1-H*	—	—	Abiotic and living cells	16	7.4	^52^
Colorimetric	AgNPs	Mentha	Mentha	Tap water	1	10.5	This work

MMT* = 5-mercaptomethyltetrazole.

1-H* = 1-[[(2-furanylmethyl)imino)methyl]-2-naphthol.

### Kinetic studies of selectivity of detection probe

**Figure 12:**
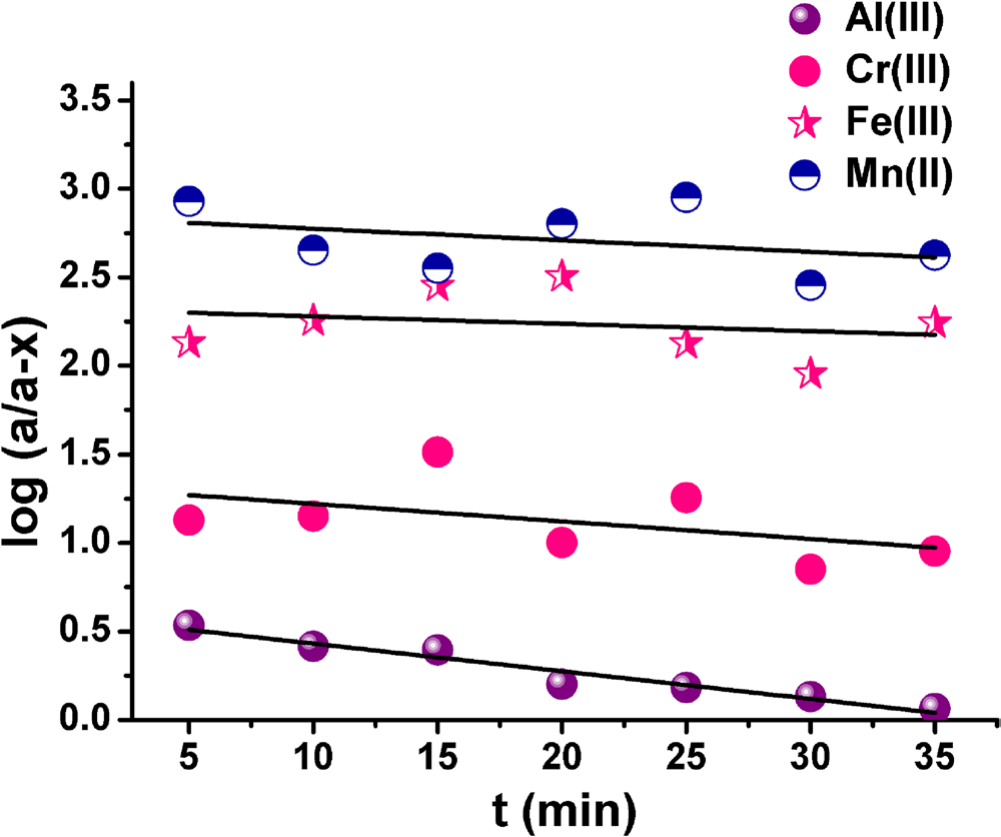
Plots of log (a/a-x) versus time (t) for kinetic study of selectivity of sensor probe with different metal ions.

Zero, first, and second-order models were applied to test the rate constant and thus elucidate the kinetics adsorption process. Of these, the reaction between probe and metal ions followed first-order kinetics having the highest value of regression coefficient (R^2^ = 0.99) (Fig. 12).

The first-order kinetic rate expression (1)

1 $$\mathrm{ln}\,a/(a-x)=-kt$$

was used to determine the values of rate constant (k) from the slope of the linear plot of $\mathrm{ln}\,a/(a-x)$ versus time (t)^56^. The plots showed the highest value of slope i.e. highest value of rate constant (Table 2) in case of the interaction between probe and Al(III). This can be attributed to the higher rate of reaction of the probe with aluminum ions, further demonstrating the selectivity of sensor towards the latter.

**Table 2:** The first-order rate constants for different metal ions.

Metal ions	Slope/rate constant (min^−1^)	R^2^
Cr(III)	0.00340	0.66
Fe(III)	0.00367	0.69
Mn(II)	0.00387	0.72
Al(III)	0.01907	0.99

### Thermodynamic studies of spontaneity of the reaction process

To reveal energetic changes and selectivity towards Al(III) as compared to other metal ions with M-AgNPs. Thermodynamic parameters of the binding process were analyzed by using equations (2–4),

2 $${{\rm{\Delta }}{\rm{G}}}^{0}=-2.303{{\rm{RTlog}}}_{10}{{\rm{B}}}_{{\rm{L}}}$$

3 $${\mathrm{log}}_{10}{{\rm{B}}}_{{\rm{l}}}({{\rm{T}}}_{2}){-\mathrm{log}}_{10}{{\rm{B}}}_{{\rm{l}}}({{\rm{T}}}_{1})=-({{\rm{\Delta }}{\rm{H}}}^{0}/2.303{\rm{R}})[1/{{\rm{T}}}_{2}-1/{{\rm{T}}}_{1}]$$

4 $${{\rm{\Delta }}{\rm{S}}}^{0}=({{\rm{\Delta }}{\rm{H}}}^{0}-{{\rm{\Delta }}{\rm{G}}}^{0})/{\rm{T}}$$

and where ∆G^0^, ∆H^0^, ∆S^0^, and B_L_ are changes in standard-state Gibbs free energy of reaction, standard-state enthalpy of reaction, standard-state entropy of reaction, and Langmuir equilibrium constant, respectively.

The results of thermodynamic parameters are summarized in Table 3, to describe the involvement of key forces contributory to the binding mechanism.

**Table 3:** Thermodynamic parameters of the detection probe.

S. No.	Metal Ions	T (K)	ΔG^0^ (kJ mol^−1^)	ΔH^0^ (kJ mol^−1^)	ΔS^0^ (J mol^−1^)
1.	${\rm{Al}}({\rm{III}})$	298	−4.224	44.56	0.1637
		303	−5.042		
		313	−5.689		
2.	${\rm{Cr}}({\rm{III}})$	298	2.531	19.501	0.0569
		303	2.246		
		313	1.965		
3.	${\rm{Fe}}({\rm{III}})$	298	2.670	24.549	0.0734
		303	2.303		
		313	2.073		
4.	${\rm{Mn}}({\rm{II}})$	298	2.744	29.044	0.0882
		303	2.303		
		313	2.073		

The negative value of ΔG^0 ^(which is highest in case of interaction with Al(III) ions) indicates the spontaneity of the binding of Al(III) ions with M-AgNPs. The positive value of ΔH^0 ^(which is highest in case of interaction with Al(III) ions) indicates that binding is endothermic. The positive value of ΔS^0 ^(which is highest in case of interaction with Al(III) ions) represents the highest electrostatic interaction between Al(III) ions and the surface of M-AgNPs as compared to other metal ions. This electrostatic force is the foremost force for the selective binding of Al(III) ions with M-AgNPs. This implies that the binding of Al(III) ions with M-AgNPs is synergistically driven by enthalpy and entropy. Whilst, the positive value of ΔG^0^ in case of Cr(III), Fe(III), and Mn(II) ions indicates the non-spontaneity of the reaction between these ions with M-AgNPs.

## Conclusions

The present study demonstrates the utilization of Mentha leaves extract as both reducing and stabilizing agent for the synthesis of AgNPs. The developed M-AgNPs present high stability in an aqueous medium and do not aggregate up to a month. The optimization of M-AgNPs for best outcomes was achieved in terms of pH, reaction time, volume of Mentha leaves extract, and temperature. The M-AgNPs presents high sensitivity from 200 to 1 nM towards Al(III). The metal ion induced aggregation of M-AgNPs causes a visual color change from light yellow to reddish-brown. The present detection method does not necessitate any surface modifying agents like DNA, thiol-containing groups or any fluorescent compounds or dyes, for metal ion detection in an aqueous system. Easy synthesis, biocompatibility and low-level detection of Al(III) ions make it an efficient probe which is easy to apply in real water systems.

## Materials and Methods

### Apparatus

Optical absorption spectra were recorded using a Lab India 3000^+^ UV-vis spectrophotometer in the range of 200–800 nm by utilizing quartz cells having 1 cm optical path length. Further, Milli−Q water was utilized as the blank for background absorption. The size and surface morphology of M-AgNPs were studied using Field Emission Scanning Electron Microscopy (FESEM, MIRA3 TESCAN), Transmission Electron Microscope (TEM, JEOL JEM-2100), and Selected Area Electron Diffraction (SAED). The mean elemental proportion of the sample was examined with Energy Dispersive X-ray (EDS) spectroscopy (Oxford instrument INCA attached to the FESEM). The surface charge and stability of M-AgNPs was examined with a zeta sizer nano ZS Malvern instrument. The FTIR measurements were recorded in the range between 4000 to 400 cm^−1^ to investigate the functional group involved in the synthesis and stabilization of nanoparticles by using FTIR spectrophotometer (Shimadzu). The concentration of metal ion was estimated by Atomic Absorption Spectroscopy (AAS). To obtain chemical state information from the topmost few nm of surface of sample XPS was used.

### Chemicals and material

All the reagents and chemicals were procured from Sigma-Aldrich Ltd and utilized without any further purification. Milli-Q water with an electrolytic conductivity of 0.055 mS cm^−1^ was used throughout the experimentation. Mentha leaves were obtained from the botanical garden of Banasthali University campus. Silver nitrate (AgNO_3_) and metal salts were obtained from Merck Pvt Ltd.

### Synthesis of M-AgNPs

Mentha (*Mentha arvensis*) leaves were washed, dried and then finally ground to powder and stirred in a 250 mL beaker with 100 mL of Milli-Q water at 80 °C for 2 h. After stirring, a dark brown solution was obtained, and the resultant extract was filtered using Whatman no. 1 filter paper (pore size 25 mm). The resultant Mentha leaves extract (MLE) was stored in a refrigerator for further application in the synthesis of M-AgNPs.

Single step synthesis of AgNPs was carried out by reducing Ag(I) ions to Ag atoms using MLE. The absorption spectra confirm the synthesis of M-AgNPs (Fig. 1). The UV-vis spectral analysis of M-AgNPs synthesized at room-temperature showed a characteristic broadband at 425 nm (Fig. 1a inset). These results demonstrate the capacity of MLE in the reduction of (Ag (I)) ions and stabilization of AgNPs.

To reduce the time of synthesis of M-AgNPs, the M-AgNPs were synthesized at 80 °C. At this temperature, a quick reduction of Ag(I) ions was achieved^57^. The AgNPs synthesized by heating method showed relatively strong and narrow UV-vis spectral peak at 419 nm (Fig. 1a inset). Such kind of blue shifting and relatively sharp spectral peak signifies that particles could be of smaller size and monodispersed. These results demonstrate that the heating method was more suitable than the room-temperature method. Therefore, M-AgNPs were synthesized by the heating method. The UV-vis spectra of M-AgNPs after a month showed no significant change in both peak position and peak intensity, thus indicating its long-term stability (Fig. 1b inset). This approved that the mentha as an effective reducing and stabilizing agent for the syntheses of AgNPs. The triplicate synthesis experiments confirmed the reproducibility of the results.

### Colorimetric detection of Al(III)

At pH 10.5 M-AgNPs was utilized in the colorimetric detection of Al(III). The different concentrations of 200 μL of Al(III) aqueous solution were added to the 400 μL of M-AgNPs solution. The solution was thoroughly mixed and kept for about 1 min at room-temperature. Finally, the resultant solution was checked for visual color change and the optical measurement carried out by UV-vis spectroscopy.

### Determination of Al(III) in tap water

The field applicability of the present study was confirmed using tap water sample of the research lab. The water sample was spiked with different concentrations of Al(III) and recovery experiment was performed. Finally, colorimetric measurement was done after 5 min.

### Kinetic and thermodynamic studies

Kinetic and thermodynamic studies were performed to confirm selectivity of detection probe and spontaneity of the reaction process, respectively. To perform these studies, some experiments were carried out, and the details of these experiments are given in supplementary information.

### Data availability

No datasets were generated or analyzed during the current study.

## Electronic supplementary material

Supplementary Information
