# Supplementary material for: Mentha-Stabilized Silver Nanoparticles for High-Performance Colorimetric Detection of Al(III) in Aqueous Systems
Source: Sci Rep. 2018 Mar 26;8:5189. doi: 10.1038/s41598-018-23469-1 (PMC5980094; doi:10.1038/s41598-018-23469-1)
Supplement: Supplementary file 1 — Supplementary Information [file 41598_2018_23469_MOESM1_ESM.pdf]

Mentha-Stabilized Silver Nanoparticles for High-Performance Colorimetric Detection of Al(III) in Aqueous Systems

Rekha Sharma[1], Ankita Dhillon[1], and Dinesh Kumar[2]

[1]Department of Chemistry, Banasthali University, Rajasthan-304022, India

[2]School of Chemical Sciences, Central University of Gujarat, Gandhinagar-382030, India

Supplementary information

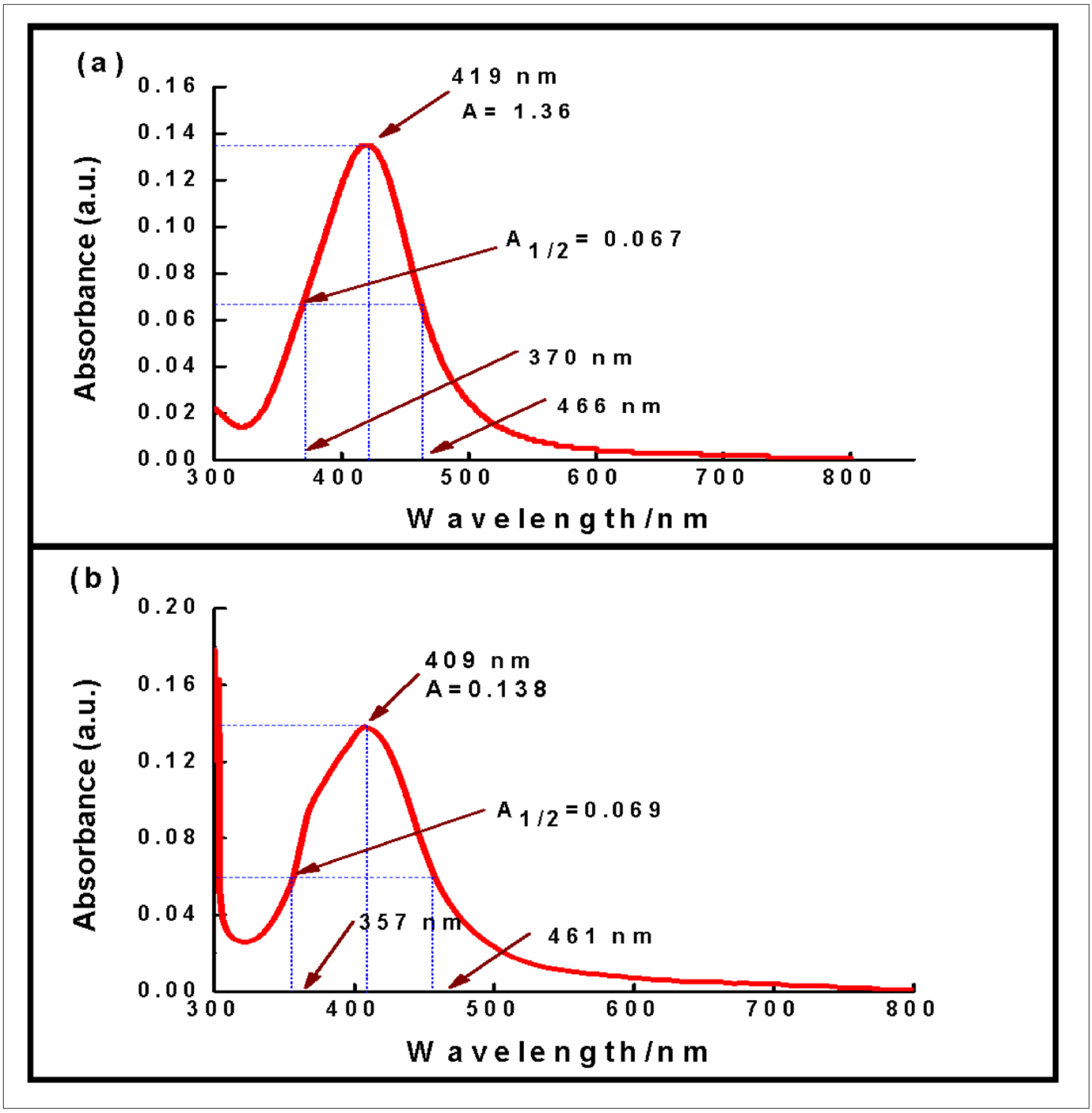

Figure S1 (a) The FWHM of M-AgNPs at 550 μL. As shown in the figure, the maximum absorbance (A) obtained at λmax= 419 nm was 1.36 and at a half maximum absorbance at the same wavelength (A at λmax1/2) was 0.68. So, the spectral width range of wavelength

extending at 0.68 was as shown in the figure with wine arrows as 370 nm to 466 nm. That means, at half maximum absorbance the full-width value would be the difference between 466 and 370 nm i.e. 96 nm. Hence, the calculated FWHM is 96 nm **(b)** FWHM value is 104 nm for extract volume 650 μL.

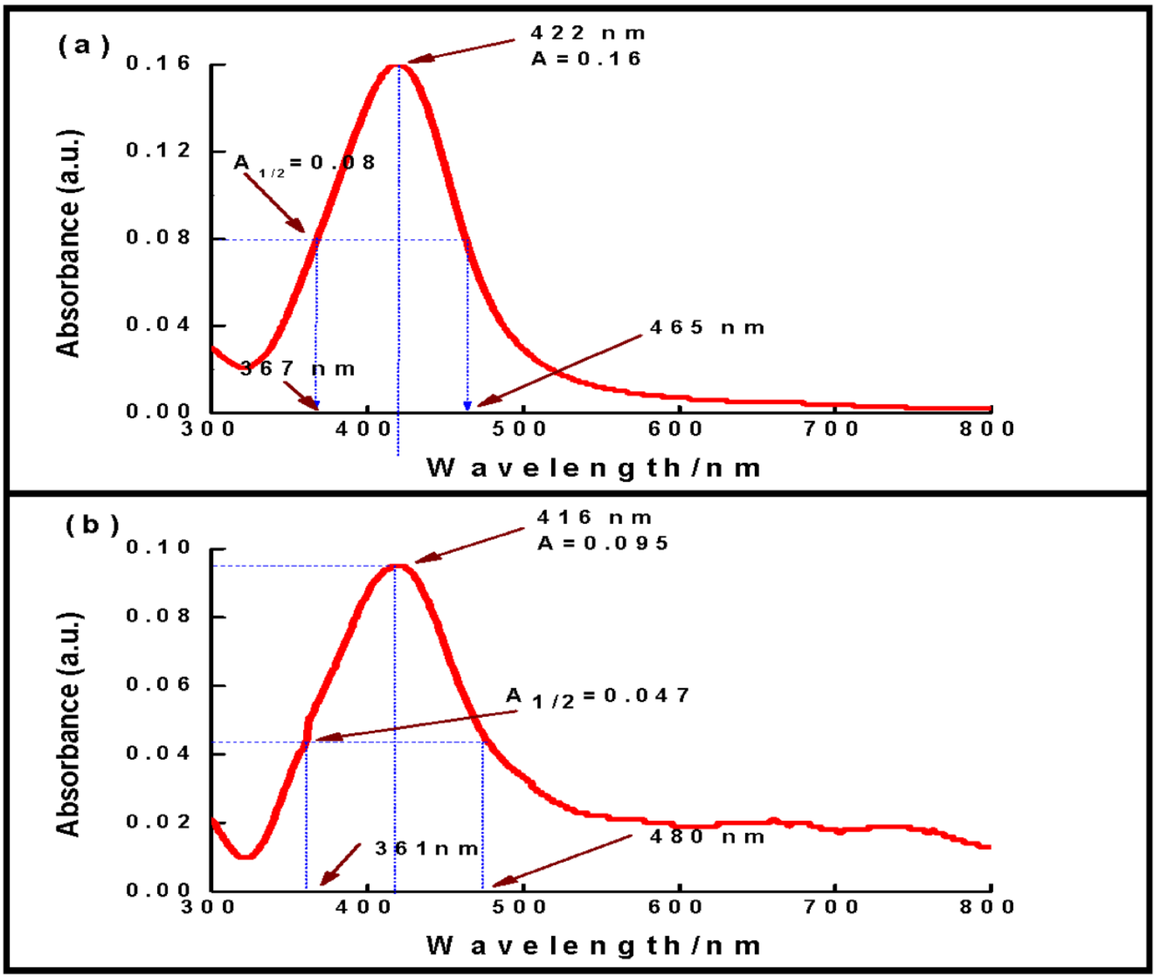

Figure S2 (a) The calculated FWHM value is 98 nm of M-AgNPs at 30 min and (b) at 40 min FWHM is 119 nm.

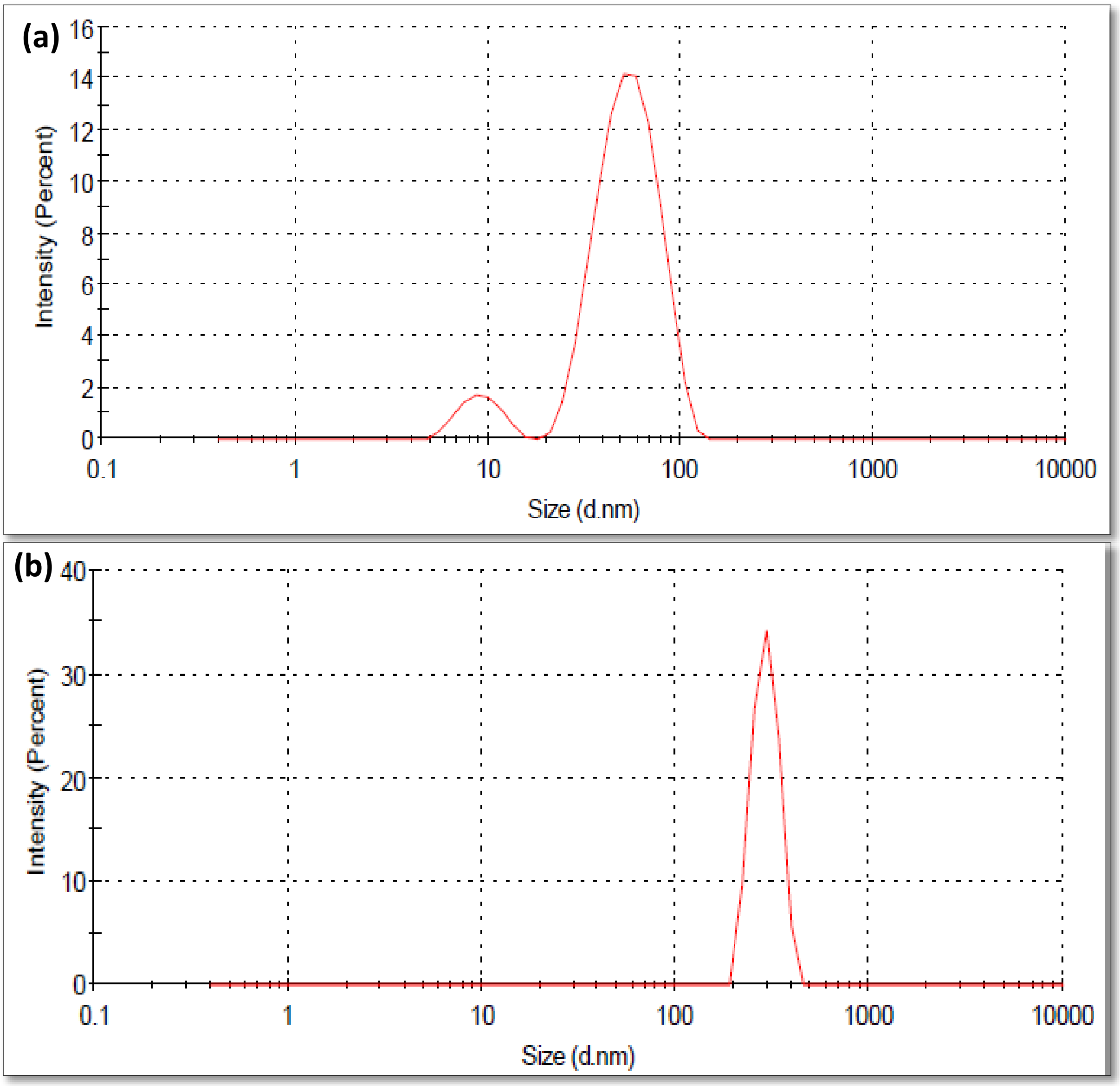

Figure S3 (a) Size distribution of M-AgNPs before and (b) after interaction with Al(III)ions.

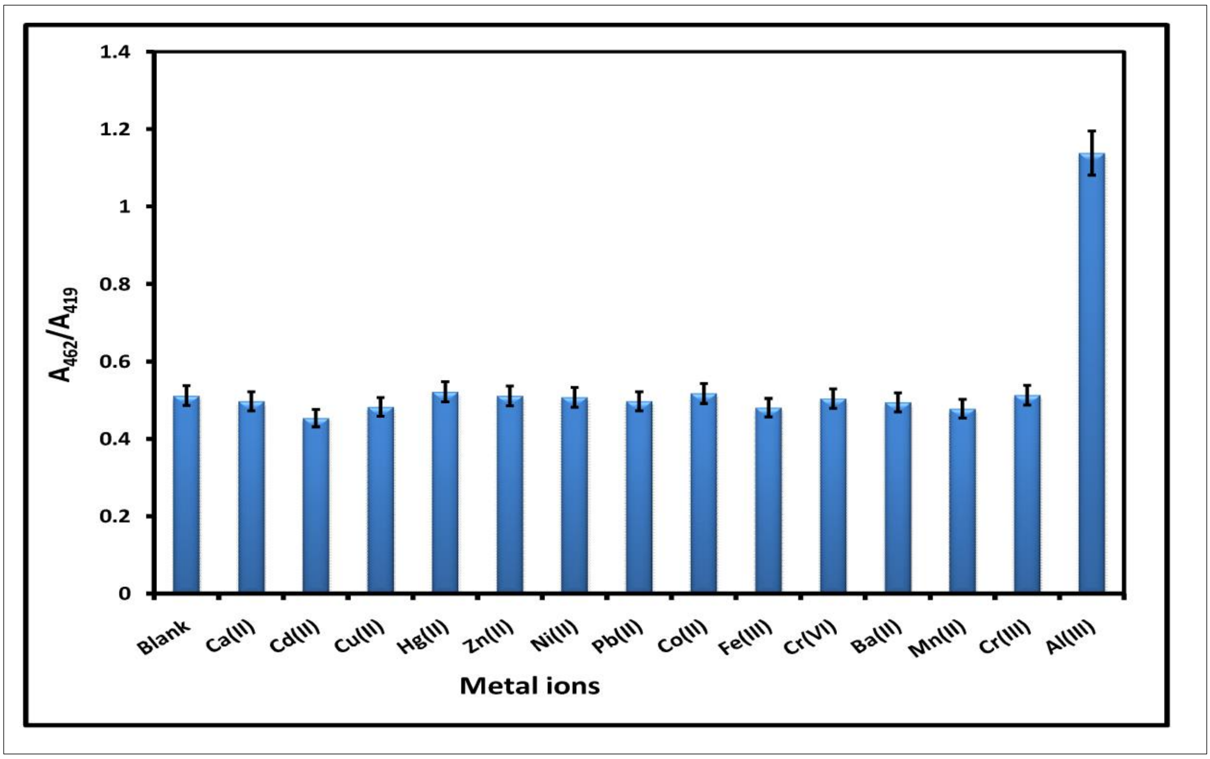

Figure S4 Bar diagram shows the specificity of the M-AgNPs based probe for Al(III) in comparison to other metal ions, error bars represent standard deviation from three repeated experiments.

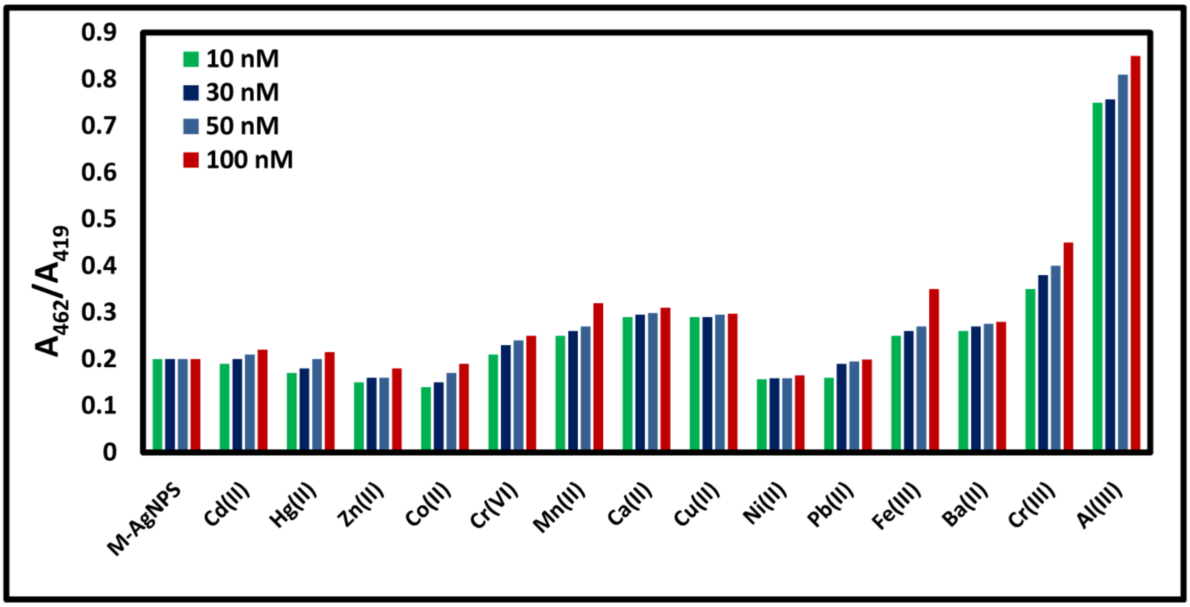

Figure S5 (a) Illustrating the selectivity of M-AgNPs in the presence of different concentrations (10-100 nM) of various heavy metal ions.

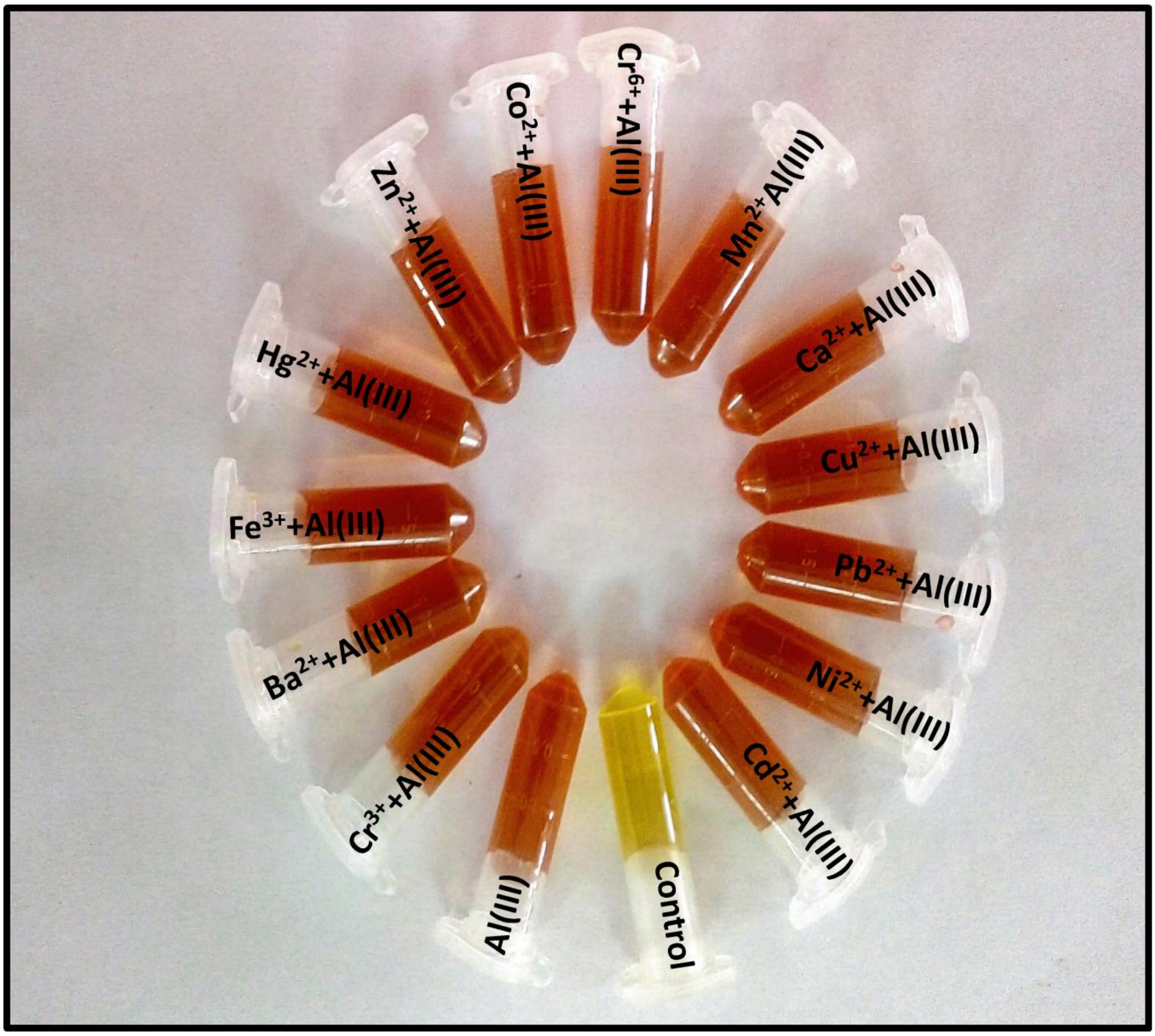

Figure S5 (b) Photographic image of the detection systems incubated with a mixture of Al(III) and other ions.

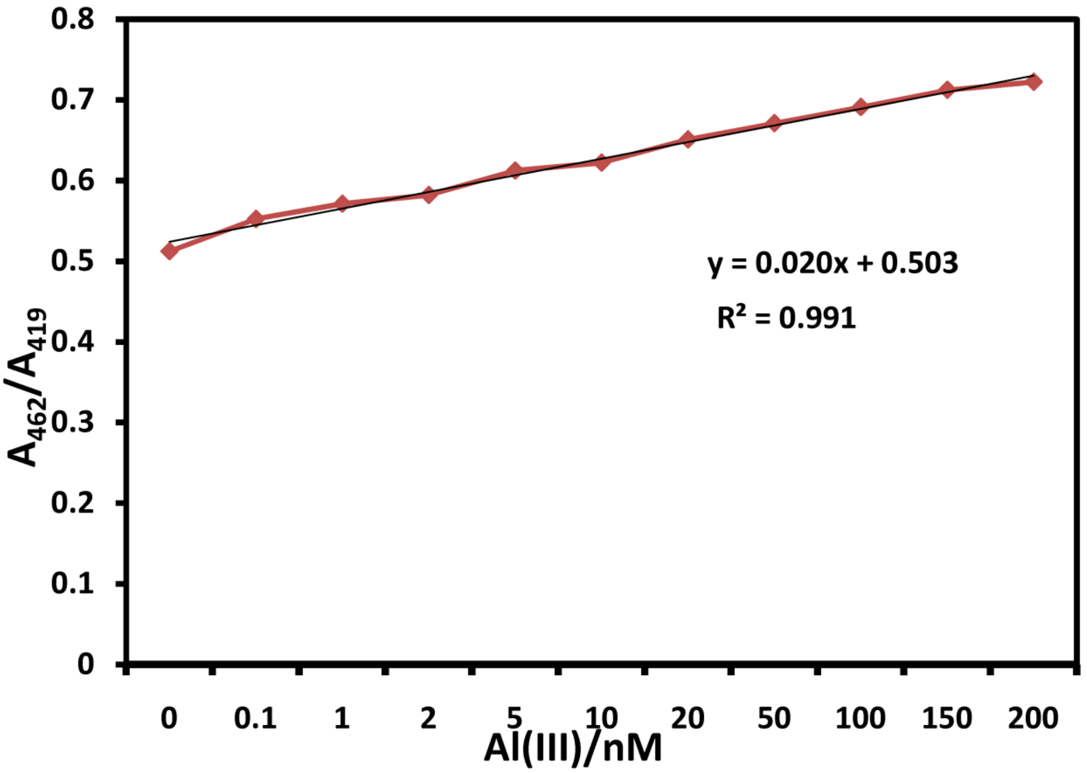

Figure S6 Plot of $A_{462/419}$ as a function of the concentration of Al(III) which shows the linearity of $A_{462/419}$ values of the M-AgNPs probe solution at 419 nm.

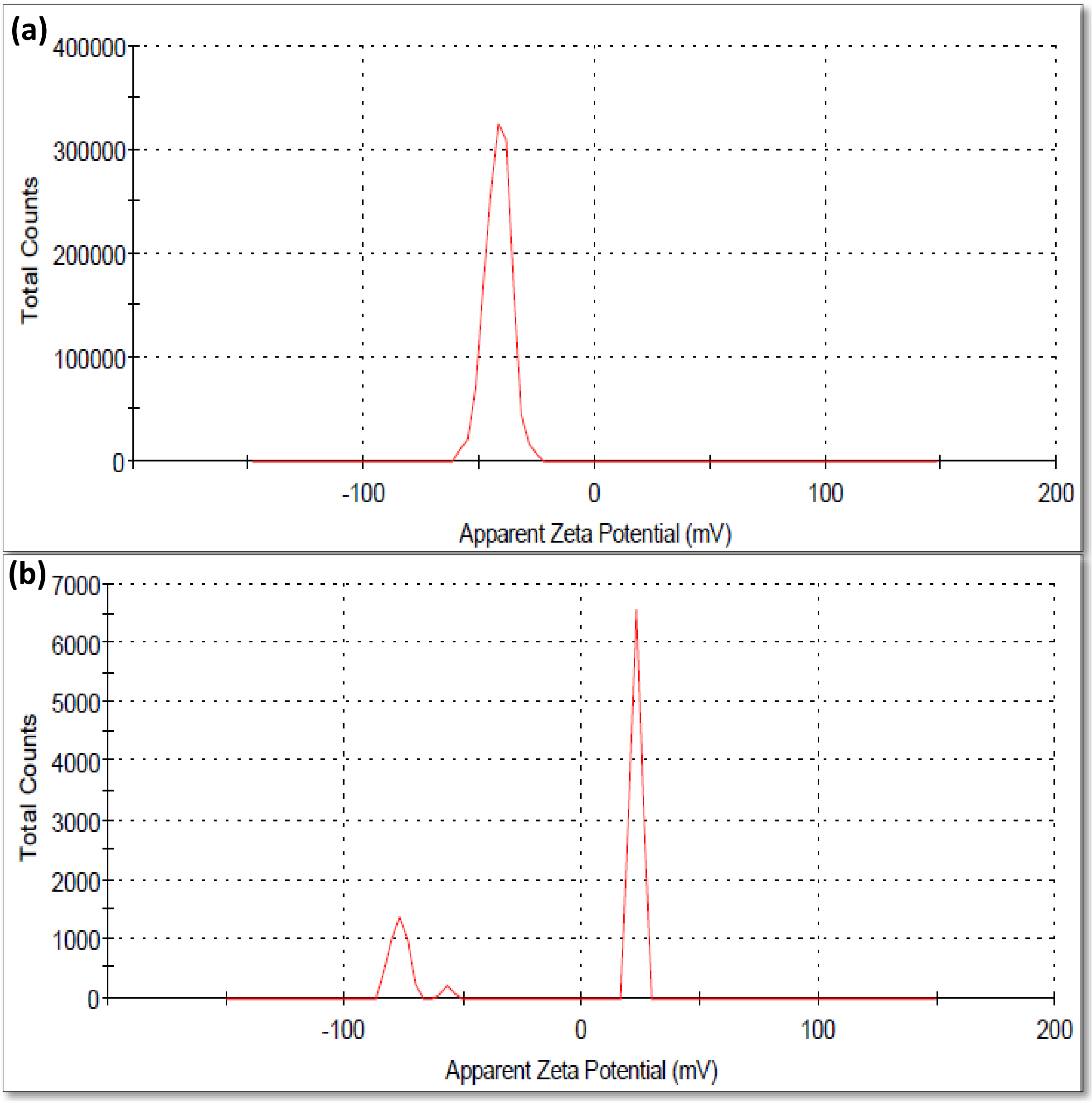

Figure S7 (a) Zeta potential distribution of M-AgNPs before and (b) after interaction with Al(III)ions.

Kinetic studies for selectivity

For the kinetic studies of selectivity of the detection probe we have taken absorbance of M-AgNPs in the presence of various metal ions such as Al(III), Cr(III), Fe(III) and Mn(II) at a concentration of 10 nM at different time interval (t) at pH 10.5 (Table 2). Then we have plotted absorbance v/s time plot to find the rate constant of the reaction process. The values of rate constant (k) were determined from the slope of the linear plot of ln $^{a}/_{a-x}$ versus t (Fig. 12).

Thermodynamic studies of spontaneity of the reaction process

In the thermodynamic experiment, 50 mL of a metal solution of a 50 nM concentration was transferred to a 100 mL vial that contained 4 mg of nanoparticles at pH 10.5. These vials were sealed and shaken continuously on a platform shaker at 250 rpm at three different temperatures such as 298, 303, and 313 K. The temperature was maintained by placing the solution on a magnetic stirrer. The solutions were stirred with a stirrer for 2 h at a pre-set temperature to reach equilibrium, the suspension was centrifuged and the equilibrium concentrations of the metal solution in the supernatant solutions were measured using atomic absorption spectrophotometer (AAS). All the experiments were performed in triplicates. The quantity of detected metal solution at equilibrium (qe, mg/g) was calculated as follows:

$$qe = \frac{(\mathrm{Co} - \mathrm{Ce})}{\mathrm{m}} \mathrm{V}$$

where C_0 (mg/L) and C_e (mg/L) represent the initial and equilibrium metal ion concentrations in solution, respectively; m (mg) is the mass of nanoparticles used, and V (mL) is the solution volume. The obtained data were studied using Langmuir isotherm model. The linear form of Langmuir isotherm is $C_e/q_e = (1/Q_0b) + (Ce/Q_0)$.

Thermodynamic parameters, such as the changes of Gibbs free energy ΔG^0, enthalpy ΔH^0 and entropy ΔS^0 for the detection of metal ions on sensor probe, are calculated wherein R is the ideal/ universal gas constant (8.314 J mol^{-1} K^{-1}), T is the absolute temperature (K), and B_L is the Langmuir equilibrium constant. The Langmuir equilibrium constant has been calculated from the slope of the linear plot 1/qe vs. 1/Ce. The results are shown in Table 3.
